# Research on Thermal Insulation Performance and Impact on Indoor Air Quality of Cellulose-Based Thermal Insulation Materials

**DOI:** 10.3390/ma16155458

**Published:** 2023-08-03

**Authors:** Cristian Petcu, Andreea Hegyi, Vlad Stoian, Claudiu Sorin Dragomir, Adrian Alexandru Ciobanu, Adrian-Victor Lăzărescu, Carmen Florean

**Affiliations:** 1 National Institute for Research & Development URBAN-INCERC Bucharest Branch, 266 Soseaua Pantelimon, 021652 Bucharest, Romania; cristian.petcu@yahoo.com (C.P.); dragomirclaudiusorin@yahoo.com (C.S.D.); 2 National Institute for Research & Development URBAN-INCERC Cluj-Napoca Branch, 117 Calea Floresti, 400524 Cluj-Napoca, Romania; adrian.lazarescu@incerc-cluj.ro (A.-V.L.); carmen.florean@incerc-cluj.ro (C.F.); 3 Faculty of Materials and Environmental Engineering, Technical University of Cluj-Napoca, 103-105 Muncii Boulevard, 400641 Cluj-Napoca, Romania; 4 Department of Microbiology, Faculty of Agriculture, University of Agricultural Sciences and Veterinary Medicine of Cluj-Napoca, 3-5 Calea Mănăştur, 400372 Cluj-Napoca, Romania; 5 Faculty of Land Reclamation and Environmental Engineering, University of Agronomic Sciences and Veterinary Medicine of Bucharest, 59 Mărăști Boulevard, 011464 Bucharest, Romania; 6 National Institute for Research & Development URBAN-INCERC Iasi Branch, 6 Anton Sesan Street, 700048 Iasi, Romania; adrian.ciobanu@incd.ro

**Keywords:** bio-based insulation material, heat transfer, resistance to micro-organisms, air quality

## Abstract

Worldwide, the need for thermal insulation materials used to increase the energy performance of buildings and ensure indoor thermal comfort is constantly growing. There are several traditional, well-known and frequently used thermal insulation materials on the building materials market, but there is a growing trend towards innovative materials based on agro-industrial waste. This paper analyses the performance of 10 such innovative thermal insulation materials obtained by recycling cellulosic and/or animal waste, using standardised testing methods. More precisely, thermal insulation materials based on the following raw materials were analysed: cellulose acetate, cigarette filter manufacturing waste; cellulose acetate, cigarette filter manufacturing waste and cigarette paper waste; cellulose acetate, waste from cigarette filter manufacturing, waste cigarette paper and waste aluminised paper; cellulose from waste paper (two types made by two independent manufacturers); wood fibres; cellulose from cardboard waste; cellulose from waste cardboard, poor processing, inhomogeneous product; rice husk waste and composite based on sheep wool, recycled PET fibres and cellulosic fibres for the textile industry. The analysis followed the performance in terms of thermal insulating quality, evidenced by the thermal conductivity coefficient (used as a measurable indicator) determined for both dry and conditioned material at 50% RH, in several density variants, simulating the subsidence under its own weight or under various possible stresses arising in use. The results showed in most cases that an increase in material density has beneficial effects by reducing the coefficient of thermal conductivity, but exceptions were also reported. In conjunction with this parameter, the analysis of the 10 types of materials also looked at their moisture sorption/desorption capacity (using as a measurable indicator the amount of water stored by the material), concluding that, although they have a capacity to regulate the humidity of the indoor air, under low RH conditions the water loss is not complete, leaving a residual quantity of material that could favour the development of mould. Therefore, the impact on indoor air quality was also analysed by assessing the risk of mould growth (using as a measurable indicator the class and performance category of the material in terms of nutrient content conducive to the growth of microorganisms) under high humidity conditions but also the resistance to the action of two commonly encountered moulds, *Aspergillus niger* and *Penicillium notatum*. The results showed a relative resistance to the action of microbiological factors, indicating however the need for intensified biocidal treatment.

## 1. Introduction

Today, worldwide, but especially in the highly and medium-developed countries, there is a change in the lifestyle of the population. On one hand, more and more activities are taking place inside buildings and, on the other hand, there is a growing awareness of the need for sustainable use of natural resources, reduced energy consumption and reduced environmental impact [1]. In this context, there is a strong orientation towards identifying new possibilities and developing new, more efficient, user-friendly and eco-friendly building materials. Various development possibilities are also being explored in the area of materials for thermal insulation of buildings, especially as the disadvantages of one of the most widely used thermal insulation materials, expanded or extruded polystyrene, are now well-known. Although it is relatively cheap, easily accessible and performs well in terms of thermal insulation, it is characterised by very low biodegradability; dangerous behaviour in the event of fire, forming burning droplets and releasing a lot of smoke; and a negative impact on indoor air quality by reducing the air and water vapour permeability of walls. In other words, it no longer allows the walls to “breathe” and leads to a “sealing” of the indoor environment, a reduction in the degree of ventilation, thus contributing to the creation of favourable conditions for the appearance and growth of mould, algae, lichens or other films of micro-organisms on the surface of the building [2,3]. This leads to a reduction in the quality of the air in the inhabited space and thus to health consequences for the population.

The literature highlights the overall negative impact on the health of users through the term “sick building syndrome (SBS)”, which is the negative manifestation of the population working, partially or totally, inside buildings affected by microorganism deposits, due to the degradation of indoor air quality through contamination with spores and toxins [1,4,5,6]. The most common mycotoxins identified in indoor air and the bodies of the population living in the contaminated environment are produced by moulds such as *Cladosporium*, *Acremonium*, *Alternaria*, *Periconia*, *Curvularia*, *Rhizopus*, *Mucor*, *Streptomyces*, *Penicillium*, *Aspergillus*, *Stachybotrys*, *Fusarium*, and *Myrothecium* [7,8,9,10], known to be genotoxic, immunotoxic, hepatotoxic, mutagenic; and potentially carcinogenic mycotoxins are ochratoxin [7] (OCT), aflatoxin B1 [9] and trichothecene [6,7,8,9,10,11,12,13,14,15,16,17,18,19].

The thermo-physical characteristics of thermal insulation materials used in the building industry include properties such as thermal conductivity, specific heat capacity, density and thermal diffusivity. These characteristics specify the efficiency of a material in terms of its ability to absorb, transfer and retain heat. Collectively, these properties contribute to a building’s energy efficiency and ability to maintain a comfortable indoor temperature, thereby reducing the need for artificial heating or cooling. The literature points to the possibility of developing materials for the thermal insulation of buildings which, in addition to their specific performance, offer some advantages in terms of opening up new opportunities for implementing the concept of the Circular Economy. However, these “alternative” insulating materials, as of 2017, only accounted for 13% of the market, and mostly comprise non-woven, fibre mattresses made from recycled plastics (the most common being polyethylene terephthalate (PET), polypropylene (PP) and polyvinyl chloride (PVC)); fibres from recycled industrial textile waste, animal or vegetable fibres (sheep wool, flax and hemp fibres, cotton wool); and/or other waste, including cellulosic waste from agricultural or industrial waste [20,21,22,23,24]. Of these, insulation materials made from agricultural raw materials, also called “bio-based insulation material”, accounted for only 6% (2012), 8% (2017) and 10% (2020) of the insulation materials market, with expectations encouraged by EU policies of 13% in 2030, of which 40–50% should be based on wood and other cellulosic materials [24]. At present, most reports in the literature focus on the methods of making such thermal insulation materials and their physical, mechanical and thermal efficiency performance, fewer on durability and very few on the impact on indoor air quality [24].

Depending on their nature, insulating materials are characterised by a thermal conductivity coefficient, λ, with values ranging from 0.024–0.07 W/mK [20]. The thermal performance of insulation made of homogeneous materials, simple or combined, is usually evaluated by the following parameters: thermal conductivity, thermal transmittance, thermal diffusivity and specific heat [25]. For the most commonly used thermal insulation materials, the current thermal conductivity coefficient range is between 0.030–0.040 W/mK for expanded polystyrene and mineral wool, 0.020–0.030 W/mK for polyurethane-based thermal insulation and 0.033–0.044 W/mK for glass fibre-based insulation [26].

Hadded et al. [23] studied recycled textiles in terms of thermo-physical characteristics (thermal conductivity and thermal diffusivity). Danihelová et al. [27] conducted a study on the performance of recycled technical textiles showing that, in line with other reports [28], mattresses made of recycled waste fibres of a vegetable or animal nature can be good thermal insulators, characterised by a thermal conductivity coefficient around 0.033 W/mK. The results of their research have shown that recycled textiles have competitive thermal properties and can be used as an alternative to the “classic” building insulation materials (extruded polystyrene or mineral wool). Thus, the thermal conductivity of these insulating materials increases with increasing temperature, identifying, for example, a thermal conductivity coefficient, λ, for recycled denim insulation that varies in the range 0.032 ÷ 0.036 W/mK in the temperature range 10 °C ÷ −30 °C and decreases with increasing density. In the same context, Valverde et al. [29] analysed the influence of the density of the thermal insulation product made by recycling textile waste on the thermal conductivity coefficient, indicating a non-linear variation, with the highlighting of a density range for which the thermal insulation performance is superior. Patnaik and Mvubua [30] created panels from layers of unspun wool, (coring wool (CW)—15 mm thick and 66.66 kg/m^3^ and dorper wool (DW)—17 mm thick and 58.82 km/m^3^), reinforced by interlacing. From the point of view of thermal conductivity, the recorded values indicate that an increase in temperature leads to an increase in thermal conductivity from 0.030 W/mK (at −5 °C) to 0.034 W/mK (at 35 °C)—for the CW sample and from 0.031 W/mK (at −5 °C) to 0.034 W/mK (at 35 °C)—for the DW sample. Zeinab et al. [28] analysed heat transfer through different types of non-woven fabrics. They studied the dependence of thermal conductivity on the thickness and density of polyester and polypropylene fibre insulation boards and concluded that, based on the measured value of thermal conductivity (approx. 0.033 W/mK), the non-woven materials analysed were suitable for use as thermal insulation material. A collective at the Brno University of Technology, Czech Republic [31] analysed the behaviour of thermal insulation boards made of recycled polypropylene R-PP and 5–20% bi-component polyvinyl chloride PVC fibres. It was concluded that in this case the thermal conductivity coefficient, λ, increases with increasing test temperature, temperature difference and density, and a product density of min. 150 kg/m^3^ provides sufficient physico-mechanical performance to allow in-situ vertical handling and positioning of the thermal insulation boards. Patnaik et al. [30] developed and analysed a proposal for a non-woven thermal insulation material based on 50% wool and 50% recycled R-PP fibres, which showed good thermal insulation performance and biological resistance.

Over time, a number of criteria have been established to assess the quality of one insulating material against another. In addition to thermal insulation performance, aspects such as impact on human health from production to end-of-life, dust or fibre emissions, biopersistence, operational safety, environmental impact, fire performance, fire toxicity, affordability in terms of price and purchase, durability and use are now being analysed. Although the development of innovative thermal insulation materials based on recycled waste would apparently solve many problems, the most important of which is the further implementation of the concept of the Circular Economy, while at the same time reducing energy consumption for indoor comfort and reducing environmental impact, a number of other challenges and difficulties arise. Thus, most of these materials, especially those developed by recovering agro-industrial wastes or by-products, are highly sensitive to water and water vapour. At the same time, because of their structure—they are often made in the form of non-woven or loose material (which requires supporting structures when put into operation)—they have a low stability of shape and dimensions. This type of material frequently weighs under its own weight, mechanical strengths are low; therefore, it is also sensitive in terms of thermal insulation performance (this is also influenced by dimensional and density aspects) [32]. Consequently, they are often conditioned by their location inside buildings, unlike the most commonly used thermal insulation materials such as expanded/extruded polystyrene or mineral wool.

Therefore, with both advantages and disadvantages, innovative thermal insulation materials, developed by recycling waste or industrial by-products, represent an area of interest with potential for exploitation, but which requires further research.

In terms of the possibilities for recycling cellulosic waste into thermal insulation materials, the advantage of these materials is that, as they are often in bulk, they can be used to insulate areas that are difficult to access for the application of other forms of insulation material (boards, panels, etc.) [32]. However, these cellulosic waste insulations, in addition to their sensitivity to water, have a very low resistance to fire, which makes it necessary to identify methods of improvement, some of which use the properties of aerogel-based composites [26]. Other studies have shown that the thermal conductivity of cellulose thermal insulation is influenced by moisture content during use, with thermal conductivity increasing with increasing moisture content. The percentage increase in thermal conductivity is higher than the increase in humidity [32]. Vejelis et al. estimate that a 1% increase in the adsorbed hygroscopic moisture content induces a 1.25–1.5%, or even 2%, increase in the λ [33,34]. As cellulose fibres are dried, their strength increases and porosity decreases, which also influences thermal insulation performance [35,36,37].

Research by Talukdar et al. [38] showed that the thermal conductivity coefficient, measured for a temperature range between 10 °C and 30 °C at an average temperature value of 22.5 °C, varies according to a polynomial function with respect to moisture content (Ø), as shown in Equation (1):

λ = (a + b·Ø + c·Ø^1.5^ + d·exp(−Ø)) (1)

where a, b, c and d are coefficients determined experimentally with the following values: a = 0.092482655, b = 0.15480621, c = 0.066517733, d = 0.1296168.

In the same trend, Sandberg [39] analysed the variation in the thermal conductivity coefficient as a function of the water absorption of the thermal insulation material, identifying a linear equation of the form:

λ = 0.037 + 0.0002·w (W/mK)(2)

where w is the amount of water absorbed per unit volume of cellulose, kg/m^3^.

In cellulosic material, water can exist in three different ways: non-freezing bound water, present in the large pores and between the fibres; non-freezing bound water, present in the mycopores of the fibre; and bound water in the hemicellulose. Experimental research has shown that capillary water tends to be lost faster than absorbed water, which induces the advantage that such materials contribute to the regulation of indoor air humidity, i.e., in low humidity conditions, they can release water; and in high humidity conditions, they can retain it [40]. However, there is a risk that under conditions of high humidity for a relatively long period, especially in cold climates, the cellulosic material may form an environment favourable to the growth of mould, which makes antifungal treatment necessary [34]. The most common antifungal treatments, which also have a role in increasing fire resistance, were those based on borax, boric acid, aluminium sulphate or ammonium sulphate, most commonly applied by wet spraying [41,42]. However, it is now known that these treatments have limited durability, may impact on human health and are not environmentally friendly; therefore, more effective methods are being sought that are resistant to accidental water infiltration and have low environmental impact and that comply with the EU Registration, Evaluation, Authorization and Restriction of Chemicals (REACH) regulation to avoid threatening human health and the environment.

From the information presented, we can identify, on one hand, the interest that the potential for revalorization of waste and industrial by-products holds in the investigation of possibilities for creating eco-innovative insulation materials. On the other hand, a series of difficulties arise due to the high degree of diversity in the type and quality of the raw material, leading to notable variations in the performance of the eco-innovative product intended for thermal insulation.

This study aimed to analyse a set of 10 types of “niche” thermal-insulation materials, produced using recycled cellulosic or agro-industrial wastes, available on the building materials market. The comparative analysis was carried out from the point of view of thermal insulation performance, simultaneously with water vapour sorption/desorption capacity and resistance to the action of moulds, all of which have implications in terms of indoor air quality. The study achieves several objectives, as follows:

- It provides a comparative analysis of the performance of a variety of thermal insulation materials that are available in the national and European construction materials market;
- The study highlights concrete possibilities for integrating waste;
- It contributes to establishing a positive environment for interdisciplinary research. This is done by simultaneously highlighting the characteristics of these materials from the viewpoint of their application field (thermal insulation of buildings), as well as the potential impact of their use on indoor air quality, and, consequently, the long-term effects on public health. This includes aspects such as resistance to fungi and other microorganism activity. This approach promotes a deeper understanding and emphasises the necessity to evaluate the performance of construction materials—in this case, thermal insulation materials—not only from the perspective of the response they provide to the requirements of their application field but also through a broader analysis. This wider analysis considers environmental impacts (such as opportunities for recycling waste, the inclusion of agricultural by-products), durability, and effects on the hygiene, safety and security conditions of the population. Historically, such analyses were mainly focused on compatibility with the field of use. However, today, in line with sustainable development strategies founded on the three core pillars (economic, social and environmental) endorsed at both European and global levels, all these requirements form an integral part of the evaluation of all materials designated for use in construction;
- Last but not least, the study seeks to improve the supportive theoretical framework. This is beneficial especially for the practical implementation of technological transfer from applied research to the production of thermal insulation materials. These materials have a high potential for recycling waste or agro-industrial by-products and are optimised from a thermal efficiency point of view and for the necessary treatments to ensure safe and hygienic use.

## 2. Materials and Methods

### 2.1. Material Characterisation

**Figure 1:**
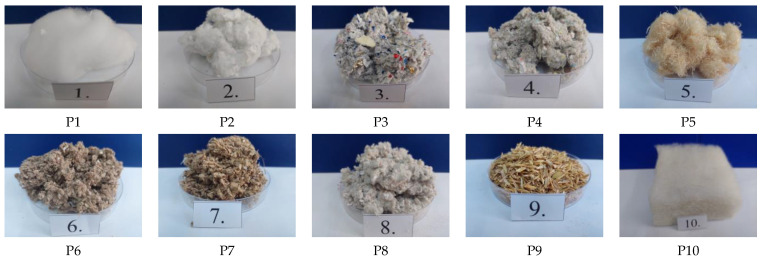
The appearance of thermal insulation materials.

The experimental investigations carried out for the comparative analysis of the performance of “niche” thermal insulation materials were conducted under laboratory conditions. From the range of materials available on the building materials market, 10 types were selected (Figure 1). Their characteristics are presented in Table 1.

While some of the selected materials might seem similar, their selection aimed to evaluate comparatively how the degree of raw material sorting and subsequent processing can influence the performances analysed. Thus, although materials P1, P2 and P3, which primarily consist of waste from cigarette filters, appear similar, it is evident that the raw material for P1 had a higher degree of sorting, specifically, just waste from these filters. In contrast, for P2 and P3, this degree of raw material sorting was reduced, with cigarette filter waste mixed with cigarette paper waste (for P2) and even with waste aluminised paper (for P3). The experimental results demonstrate that their thermophysical characteristics are quite distinctive, also. Comparing these three situations could be particularly beneficial for cigarette manufacturers seeking to optimise their waste recycling process, and independent production units, which could impose certain conditions on the raw material they acquire for producing such thermal insulation materials. For similar reasons, materials P4 and P8 were selected. Although they seem to use raw materials from the same waste category (waste paper), they are produced by two different manufacturers, and specific elements of the technological process could influence the final product. Conversely, materials P6 and P7, manufactured by the same entity and derived from similar waste materials (waste cardboard), are subject to varying degrees of technological processing. Therefore, the experimental results for P6 and P7 are particularly beneficial for manufacturers of construction materials. They increase awareness regarding performance and show a high level of product quality is required to ensure the desired energy efficiency and hygiene in the occupied spaces. Despite their similarities or differences, all these selected materials have a common denominator: the cellulose component in various forms.

### 2.2. Assessing the Impact of Density and Humidity Content on Thermal Conductivity

The thermal insulation efficiency of the products was evaluated using the thermal conductivity coefficient λ (W/m·K). Thermal conductivity was measured with a specialised instrument, the λ-Meter EP500e guarded hot plate equipment (Lambda-Messtechnick GmbH, Dresden, Germany). The entire process was carried out following the guidelines of the SR EN 12667 [43] standard. This approach allowed for a complete characterisation of the products based on their thermal insulation efficiency.

For our experimental investigation, we selected test temperatures of 10 °C and 23 °C and subjected the materials under test to specific humidity conditions (dry material and material conditioned at 50% relative humidity). These choices were made in compliance with the SR EN ISO 10456 standard [44], which explicitly outlines this set of conditions for reporting the values derived for the thermal conductivity coefficient.

**Figure 2:**
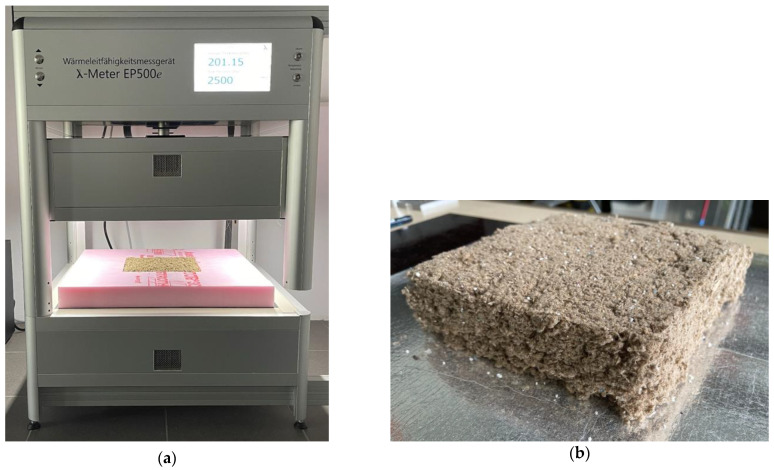
Testing the P6 material: (**a**) The setup used for determining thermal conductivity at the specified density; (**b**) The product, after testing it at maximum density and following extraction from the XPS frame.

Testing of bulk materials and loose insulation typically involves the use of incompressible frames [45,46,47], often made of polyurethane (PUR) or extruded polystyrene (XPS). These materials are not only more durable for laboratory tasks, but they also prevent mass transfer from the external environment due to their sealed pore structure. For this study, XPS frames with predetermined volume were used. Each frame measures 500 × 500 × 50 mm on the exterior to fit λ-Meter EP500e plates and features a rectangular cavity measuring 200 × 200 × 50 mm where the materials were tested, as is shown in Figure 2. This approach ensures that the testing area, which on λ-Meter EP500e measures 150 × 150 mm [48], exclusively comprises the material under test, eliminating any possibility of inadvertent overlap with the frame. The frame is positioned in the guarded area of the equipment, ensuring that the sample is tested at the specified density. The sample is shielded from external factors such as humidity, and there is no interference with the heat flow generated by the equipment [48]. This heat flow is used to determine the sample’s equivalent conductivity using an absolute measurement technique applicable to samples of a rectangular shape [49].

While adherence to certain standards is not necessarily mandatory from a research perspective, complying with these established guidelines ensures our results are reproducible and can be independently validated. Moreover, the results could be useful during potential revisions of the standards. Therefore, tested material was conditioned following standard SR EN 15101-1+A1: 2019 [50] in an oven at a temperature of (70 ± 2) °C, using air from the laboratory at (23 ± 2) °C and (50 ± 5)% relative humidity. The testing procedure started with identifying the natural density of the product, defined as the lowest density at which the material could sustain its form under its own weight. Every test is done for three average temperatures of the sample: (10 ± 1) °C, (23 ± 1) °C and (40 ± 1) °C. Following the initial conductivity test with the dry material, it was conditioned using a climate chamber Angelantoni Challenge CH250 (Angelantoni Industrie Srl, Massa Martana, Italy), at a temperature of 23 °C ± (0.25 ÷ 0.3) °C and a relative air humidity (RH) of (50 ± 1)%. The conditioning time was specific to each type of material analysed, in order to achieve constant mass. Constant mass was considered reached when, between two successive weighings, there was no difference greater than 0.1%. This way of testing was considered necessary because it is known that humidity influences thermal conductivity performance [44,51]. After constant mass was achieved, the product was retested, using the same protocol, at (10 ± 1) °C, (23 ± 1) °C and (40 ± 1) °C.

Another stage in the assessing of the thermal efficiency of the materials involved studying the change in the thermal conductivity coefficient relative to density. To achieve this, after the sample was examined in both dried and normal conditions, extra material was incrementally added to the frame until the necessary density for the next test was reached. The extra material was introduced in uniformly distributed layers. Each layer was compacted before the addition of a new layer, in order to ensure a consistent sample density throughout. This approach to analysis was considered important for two primary reasons: firstly, due to the significant impact a material’s density can have on its thermal insulation properties, and secondly, due to the high likelihood of the material compacting under its own weight. The final stage of evaluating the materials’ thermal performance analysed how the thermal conductivity coefficient varied in relation to both density and moisture content. To do this, the test samples, once dried to a constant mass and tested for a given density, were placed in a climate-controlled chamber at a temperature of 23 °C ± (0.25 ÷ 0.3) °C and a relative humidity of (50 ± 1)% until they achieved a constant mass, after which the thermal conductivity coefficient was determined for the sample conditioned in this way. Due to existing compaction, it was observed that the moisture permeates the material more slowly. As a result, the conditioning period required for the samples to attain a constant mass was longer than it was for samples at their natural density.

### 2.3. Characterisation of Materials in Terms of Hygroscopicity Performance

The hygroscopicity characteristics were evaluated by analysing the sorption/desorption curves according to the methodology indicated in European standard SR EN ISO 12571:2021 [52]. The sorption/desorption capacity of water vapour was quantified by plotting the characteristic curves of the variation of the specimen mass as a function of the relative air humidity, w(%) = f(RH(%)). For testing, specimens were exposed to the ambient environment in a closed room at a constant temperature (23 ± 0.5) °C, varying only the humidity parameter (RH) with an accuracy of ±1%. The climatic chamber used was of the type FDM F.Iii Della Marca C700BXPRO RT100 (F.lli Della Marca s.r.l., Rome, Italy), with a temperature accuracy of ±0.5 °C and a humidity accuracy of ±1%. Initially, for sorption curve plotting, the relative humidity was increasing in 5 steps, 30%, 45%, 60%, 75% and 90%, respectively. Subsequently, for the analysis of the moisture desorption capacity, the air humidity variation plot was plotted in reverse, also in 5 steps, 90%, 75%, 60%, 45% and 30% respectively. For stabilisation of the monitored parameters, the specimens were kept at each RH value for 7 days, after which they were weighed to an accuracy of 0.0001 g. To ensure repeatability conditions, 5 determinations were performed for each test, presenting the mean value of the results.

### 2.4. Characterisation of Materials in Terms of Resistance to the Action of Micro-Organisms

The resistance to the action of micro-organisms was carried out in two test variants: in the case of exposure of thermal insulation materials under conditions of high humidity and under conditions of an environment contaminated with micro-organisms, respectively.

#### 2.4.1. Analysis of the Risk of Mould Growth When Thermal Insulation Materials Are Exposed to High Humidity Conditions

In the first testing variant, in accordance with the working methodology indicated in the European guide on the elaboration of technical agreements for the marketing of construction products in accordance with European regulations, EAD 040138-01-1201 [53] and EAD 040005-00-1201 [54], and the European standard SR EN ISO 846:2019 [55], the test specimens were exposed in an environment with high humidity achieved as a result of water evaporation under conditions of normal pressure and constant temperature (23 ± 1) °C, in a closed enclosure. This method is specific to the evaluation of the biological resistance of thermal insulation materials or acoustic insulators made of animal fibres, manufactured in the factory or in-situ. During the 28 days of exposure, the specimens did not come into contact with liquid water. At regular intervals (5, 10, 15, 20 and 25 days) and at the end of the 28 days of testing, the test specimens were examined visually without optical magnification and microscopically using a LEICA SAPO microscope (Leica Microsystems, Wetzlar, Germany) to identify signs of mould growth. The quantification of resistance to the action of microorganisms was evaluated by classifying the fungal growth in rating classes and categories indicating the performance of the product in terms of nutrient content conducive to the growth of microorganisms, according to the SR EN ISO 846:2019 [55] reference, summarised in Table 2. With the character “+”, it was evidenced that the fungal load increased significantly from the evaluation performed at exposure time t to the evaluation performed at exposure time t + 1, without however being a sufficient change in order to affect the fungal growth rating class.

#### 2.4.2. Evaluation of the Resistance of Thermal Insulation Materials in Conditions of a Micro-Organism Contaminated Environment

In the second variant of testing the resistance of thermal insulation materials to the action of moulds, systems of exposure of specimens in an environment contaminated with two species of moulds, most encountered in daily activity, were carried out. The microbial laboratory procedure was performed on potato dextrose agar (PDA) culture media in the presence of two fungal species—*Penicillium notatum* and *Aspergillus niger*. Each sample of insulation was placed in the middle of a Petri dish, and the two fungal species were inoculated on media in a cross-system. Both species were collected from the indoor walls of buildings and were chosen as fungal contaminants due to their presence in inhabited areas. Inoculation was performed with a sterile 10 µL loop and the fungal-insulation culture was incubated at 25 °C for 5 days. A secondary culture test was performed, with insulation incubated on culture media. The test was designed to assess the fungal component present on insulation. The readings were performed each 24 h with the recording of results on the 6th level of the proposed scale.

## 3. Results and Discussion

The results of the experimental research on thermal insulation performance including water vapour sorption/desorption capacity and resistance to mould growth, all of which have implications for indoor air quality, showed similarities and differences between the 10 types of materials analysed. These specific behaviours of each material type are believed to have been influenced by several factors, of which the characteristics of the raw material used to make the material is the main one.

### 3.1. Characterisation of Materials in Terms of Thermal Insulation Efficiency

**Figure 3:**
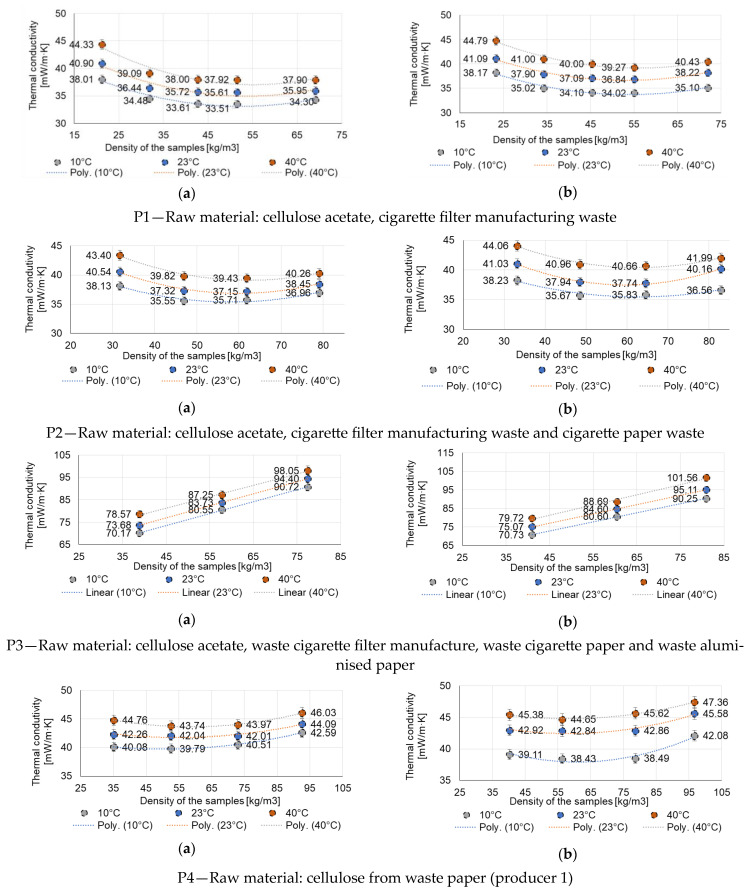

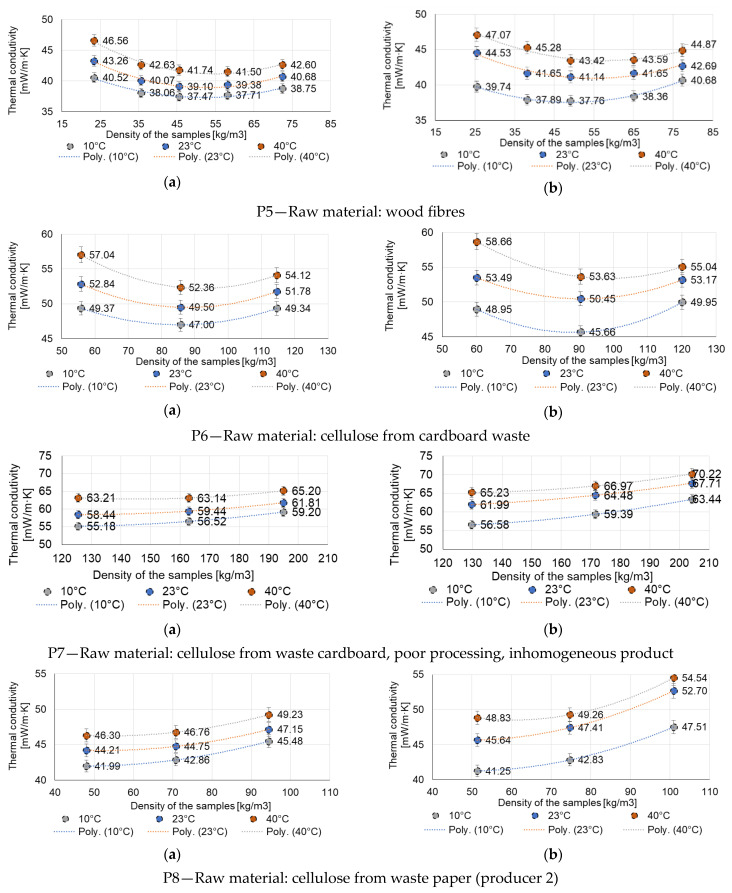

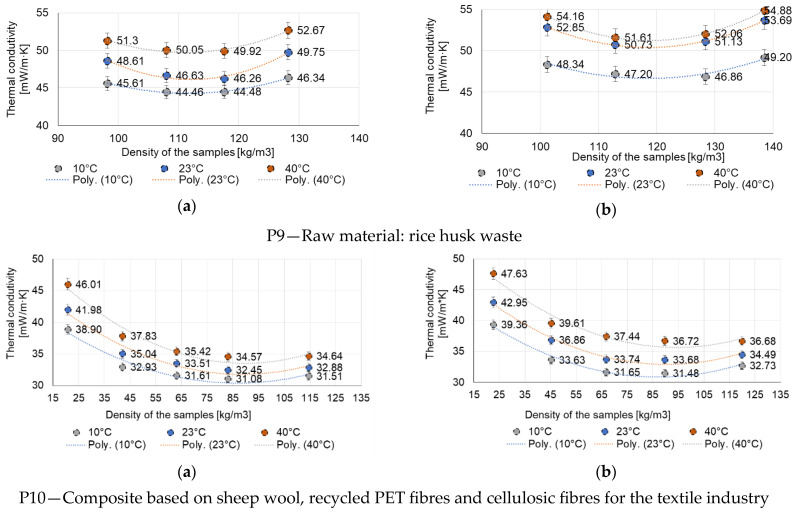
Influence of material density: dry (**a**), respectively, conditioned at 23 °C ± (0.25 ÷ 0.3) °C and RH (50 ± 1)% (**b**), on the thermal conductivity coefficient determined at sample average temperature of 10 °C, 25 °C or 40 °C.

Heat transfer through a solid sample of material primarily occurs through conduction and convection mechanisms, as is already established [56,57,58,59]. Convection is affected by the arrangement and volume of air gaps between particles [60,61], while conduction is influenced by the contact area and number of contact points between solid particles [62]. These mechanisms collectively determine how heat is transferred. As the density of a material increases due to subsidence, the size of air voids decreases and the number of contact points between particles increases. Therefore, when compressing a uniformly air-filled material, the convective component of the heat transfer is reduced, while the conduction is increased due to improved connectivity between the solid parts [63,64]. For thermal insulators that incorporate recycled textile fibres [25,29] or natural fibrous insulation material, byproducts from the agricultural industry [60,64], polynomial correlations between thermal conductivity and density have been already presented. The test results from the present research indicate that compression of a low-density product with uniformly distributed air gaps leads to a significant decrease in thermal conductivity, as seen for the products P1, P2, P5, and P10 in Figure 3. Compression produces a sharp decline in convective heat flow in the initial segment of the density range, specifically between the natural density of the material and approximatively 40 kg/m^3^. For this interval, the reduction in convective heat flow surpasses the increase in the conductive part of heat transfer. However, after a certain density threshold is reached, the rate of increase in heat transfer through conduction surpasses the decrease in convection; therefore, the value of thermal conductivity rises. For easier comparison of the evolution of the thermal conductivity coefficient depending on density, for the dry situation (a), versus conditioned at 23 °C ± (0.25 ÷ 0.3) °C and RH (50 ± 1)% (b), the graphical representation for each of the analysed thermal insulation materials was made while maintaining the same scale (on the X and Y axes).

Increasing material density leads to different variations in thermal conductivity, depending on the type of material. Thus, in the case of samples P1, P2, P10, the increase in density initially causes a reduction in the thermal conductivity coefficient, with a tendency to linearise, possibly with the recording of a minimum value on the graph λ = f(density) marked by an inflection point. A similar trend, with the existence of an inflection point on the λ = f(density) graph, but without a linearisation tendency, i.e., with the existence of an inflection point, is also recorded for the materials P4, P5, P6, P9. It should be noted, however, that in these cases the zone of decrease and existence of the inflection point is more evident, followed by a zone of increase in the thermal conductivity coefficient with increasing density. This trend is particularly evident in dry samples. Testing these materials after they have been conditioned at 50% RH, the λ = f(density) variation graph undergoes changes, generally emphasising the existence of the inflection (minimum) point. Therefore, it is considered that, in the case of these materials, increasing the density to a material-specific value may have beneficial effects by reducing the thermal conductivity coefficient. Above this optimum density value, the effect on the coefficient of thermal conductivity is either not significant (λ increases by less than 10%, e.g., sample P1) or even negative (λ increases by more than 10%, e.g., samples P2, P4, P6).

An entirely different aspect is presented by the λ = f(density) curves for samples P3, P7 and P8. In the case of these materials, an increase in the thermal conductivity coefficient is clearly identified as the density increases, both for tests on dry samples and material conditioned at 50% RH. Therefore, in these cases a densification of the material, either as a result of compaction under its own weight or by the way it is laid, induces disadvantages in terms of thermal insulation performance, all the more evident as the temperature difference, warm zone-cold zone, is greater (more obviously identified on the graph of sample P8 conditioned at 50% RH where the distance between the λ = f(density) curve recorded at the 10 °C test temperature is far removed from the λ = f(density) curve recorded at 23 °C).

A comparative analysis of the values recorded for each dry tested material, respectively, after conditioning at 50% RH, shows that, in general, the existence of moisture content in the material leads to slight increases in the thermal efficiency indicator. Exceptions are recorded in the case of testing at 10 °C for samples P3, P4, P5, P8.

Increasing the test temperature of the material (10 °C, 23 °C or 40 °C) generally results, for each material analysed, both dry and conditioned at 50% RH, in a similar appearance and parallel positioning of the λ = f(density) graphs; the higher the test temperature, the higher the thermal conductivity coefficient. Therefore, it is estimated that the benefit obtained by using such materials is greater the lower the temperature, which contributes to obtaining a benefit on the quality of indoor comfort and energy consumption, especially in cold climates.

By analysing the thermal performance indicator, λ, as a function of the macroscopic structure of the material, some similarities in behaviour can be identified. For example, samples P1, P2 and P5, cellulose thermal insulation materials with a fibre-bound appearance and even P10, which is a fibre composite, show λ = f(density) graphs with a similar appearance. Therefore, it is considered that the type, raw material, structure, density and moisture content are important factors influencing the thermal insulation performance, being in correlation with previous studies [38].

The functions showing the variation of the thermal conductivity coefficient with respect to density are shown in Table 3, and those showing the variation of the coefficient of thermal conductivity with respect to the mean temperature of the samples during the test are shown in Table 4. It should be noted that, in order to achieve a correct degree of appreciation of the variation function, the condition was imposed that the correlation factor R^2^ > 0.9. On the basis of these functions, at value 0 of the derivative of the function, it is possible to determine the density value for which the thermal conductivity coefficient is minimum, as shown in Table 5. Thus, it can be said that an increase of 149.1% (P1), 83.9% (P2), 41.6% (P4), 116.0% (P5), 54.3% (P6), 5.8% (P9), 325.1% (P10), respectively, will lead to a reduction of the thermal conductivity coefficient recorded on dry material, at 10 °C, λ_10,ct._, by 13.2% (P1), 7.4% (P2), 0.7% (P4), 8.2% (P5), 5.2% (P6), 3.5% (P9), 22.4% (P10), representing the highest benefit that can be obtained in this way. As can be seen, in some cases, this effort to increase the material density is substantially beneficial and justified; in other cases, it is less significant in terms of improving thermal performance. An exception is recorded for the P3 material for which the identified function is linear, continuously increasing. In this case, no benefit of reducing thermal conductivity can be obtained by increasing the material density; the λ_10,ct._ value recorded experimentally at natural density being the lowest of the set of experimental values. Similarly, the P8 material is identified as an exception whose density, theoretically, should be reduced by 4.4% in order to achieve an optimal conductivity coefficient λ_10,ct._ Although these theoretical conclusions exist, reducing the natural density of the materials (case P3 and P8) is hardly possible from a practical, implementation point of view, possibly requiring interventions in the manufacturing technology. Another exception was the P7 material, for which, although apparently polynomial functions were identified, it turned out that the evolution of the λ = f(density) curve is increasing and with the increase in the density of the material, the thermal conductivity coefficient also increases, the subsidence even under its own weight having a negative effect. In fact, of all the materials analysed, P7 is considered to have the lowest quality, being inhomogeneous, with frequent agglomerations of material, which could be one of the explanations for this behaviour: not only the type of source material but also the quality of its processing, the homogeneity of the final product, have a great influence on the thermal insulation performance.

By comparing the experimentally obtained thermal conductivity coefficient of the materials with general values characteristic of commonly used thermal insulation materials or cellulose-based thermal insulation materials reported in the literature [32] (Table 6), it is judged that in terms of efficiency as an insulating material, P1–P10 materials correspond to the intended field of use.

These findings align with similar results documented in the specialised literature [25,46,60,65,66,67].

### 3.2. Characterisation of Materials in Terms of Hygroscopicity Performance

**Figure 4:**
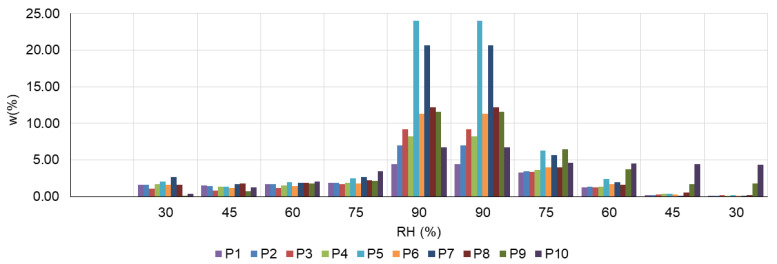
Average amount of adsorbed/desorbed water, expressed as a percentage of the constant mass of the specimen, recorded at RH of 30%, 45%, 60%, 75% and 90% during the sorption/desorption cycle.

**Figure 5:**
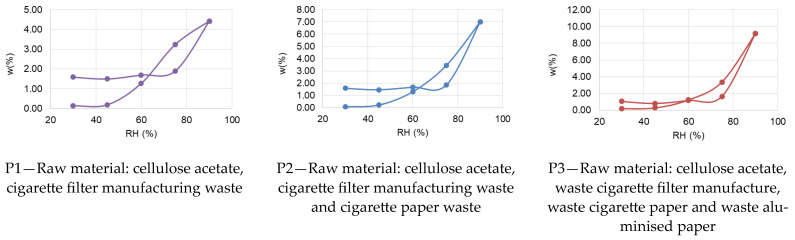

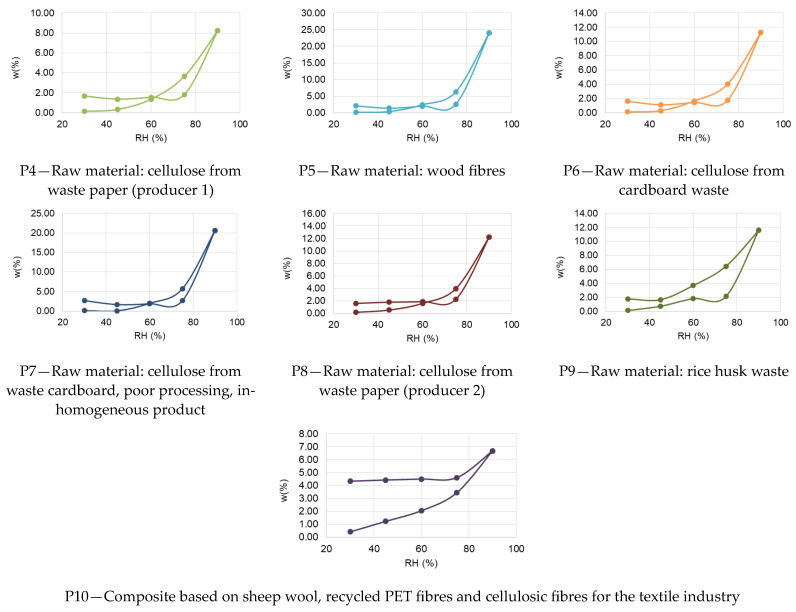
Sorption/desorption curves recorded for thermal insulation materials.

The hygroscopicity of the thermal insulation materials evaluated was analysed using as a measurable indicator the variation of the specimen mass as a function of relative air humidity, w(%) = f(RH(%)). The average amount of adsorbed/desorbed water, expressed as a percentage of constant specimen mass, for each material type is shown in Figure 4; the sorption/desorption curves are shown in Figure 5.

Analysing the evolution of the indicators presented in Figure 4, it can be said that, in general, for all the 10 types of material analysed, there is a relative constancy of the sorption phenomenon, with a slightly increasing trend, up to and including RH 75%. When the specimens are exposed to RH 90%, the sorption phenomenon is greatly amplified for all materials, with the most evident intensification of sorption being recorded for P5 and P7 materials. Subsequently, going through the relative humidity decrease diagram induces, as expected, the desorption phenomenon in each material type. In this area, a hindrance of water loss is noticed at RH 75%. Specifically, it is shown that materials exposed to RH 75% in the desorption zone (gradual RH decrease) store more water than the same specimens in the situation where they have crossed the sorption diagram (gradual RH increase). Therefore, part of the water adsorbed at maximum RH (90%) is no longer delivered to the environment when RH is reduced to 75%. A similar phenomenon occurs for samples P9 and P10 in the RH 60–30% range. For all the other materials, in the desorption zone at RH 60%, 45% or 30%, there is a strong reduction of stored water, i.e., a very good capacity to release water as the relative humidity decreases.

Analysing the positioning of the sorption/desorption curves with respect to each other (Figure 5), for the case of P1–P8 materials, the existence of an intersection point is observed each time. Initially the sorption curve has a relatively linear evolution and is placed above the desorption curve. In the vicinity of relative air humidity RH = 60% (with small variations from one material to another) and up to RH = 90%, the position of the curves changes, the desorption curve being placed above. This behaviour indicates the strong influence that the relative humidity has on the ability of these materials to regulate the humidity of the indoor environment. On the other hand, the behaviour of P9 and P10 materials is different. In these cases, the intersection of the two curves, sorption and desorption, is no longer recorded, the sorption curve being placed below the desorption curve. This behaviour indicates the limited capacity of these materials to release the stored water in order to contribute to the regulation of this parameter under conditions of reduced relative humidity of the air in the interior space.

By analysing the behaviour of all 10 material types, it can be hypothesised that the type of material and its characteristics significantly influence its ability to contribute to air humidity regulation (indoor air quality parameter).

On the other hand, according to the literature, due to the limited capacity to release adsorbed moisture, there is a possibility of accumulation of this moisture in the insulation material. This accumulation of moisture not only does not help to regulate the humidity of the air in the interior space but may even lead to the creation of an environment favourable to the growth of mould, as shown in studies carried out mainly in northern climates [43,44,45,68,69,70]. Therefore, considering that residual humidity contributes to creating a suitable environment for the development of microorganisms, as presented in Section 3.2, it is considered that the analysed thermal insulation materials would be more suitable for use for the function of thermal insulation rather than for the function of regulating the humidity of the air in the interior space, by applying a vapour diffusion barrier to avoid mass transfer to the thermal insulation layer.

### 3.3. Characterisation of Materials in Terms of Resistance to the Action of Micro-Organisms

As the samples of materials analysed were taken from the footprint of the building materials, it is not possible in this case to analyse whether the existence of an antifungal treatment influences their behaviour. The existence of this antifungal treatment, as well as details of the treatment solution, is specific to each manufacturer and often confidential information, which provides elements that contribute to the degree of competitiveness among the manufacturers of these niche materials.

#### 3.3.1. The Risk of Mould Growth If Thermal Insulation Materials Are Exposed to High Humidity Conditions

**Figure 6:**
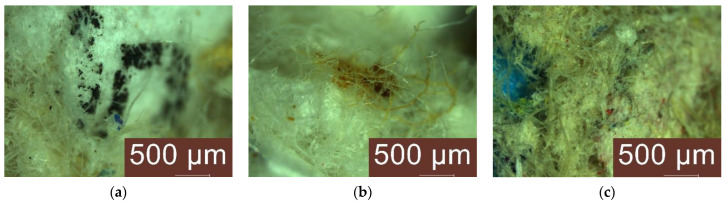

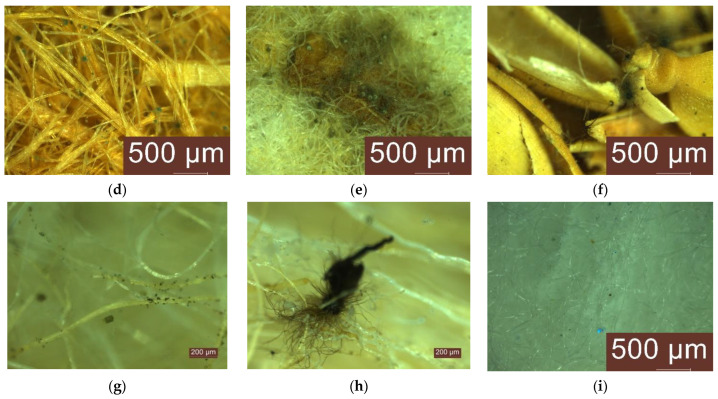
Example images of microscopic mould identification. (**a**) P8 (cellulose from wastepaper, producer 2)/10 days, (**b**) P3 (cellulose acetate, waste cigarette filter manufacture, waste cigarette paper and waste aluminised paper)/20 days, (**c**) P4 (cellulose from wastepaper, producer 1)/15 days, (**d**) P5 (wood fibres)/28 days, (**e**) P2 (cellulose acetate, cigarette filter manufacturing waste and cigarette paper waste)/28 days, (**f**) P9 (rice husk waste)/28 days, (**g**) P10 (composite based on sheep wool, recycled PET fibres and cellulosic fibres for the textile industry)/15 days, (**h**) P10 (composite based on sheep wool, recycled PET fibres and cellulosic fibres for the textile industry)/28 days, (**i**) P1 (cellulose acetate, cigarette filter manufacturing waste)/28 days.

Visual and microscopic analysis of thermal insulation materials made by recycling selected agro-industrial wastes were carried out after 5 days, 10 days, 15 days, 20 days, 25 days and 28 days, respectively, of exposure under conditions of high humidity and constant temperature. The experimental results are shown in Figure 6 and summarised in Table 7 by indicating the rating class of fungal growth and performance category in terms of nutrient content conducive to the growth of micro-organisms, according to SR EN ISO 846 [55].

Visual analysis, without optical magnification means, indicates a superficial degradation of the materials; they show slight changes in appearance, mostly in terms of colour, with a “slightly moist and damp” appearance.

Microscopic analysis of the materials showed signs of mould growth from the evaluation after exposure to damp conditions for 5 days in the case of samples P3 and P4, and from the evaluation after 10 days in the case of samples P2, P6, P8, P9. Evaluation after exposure for 15 days in a wet environment showed the presence of mould in all samples except P1. Moreover, in this sample P1, increased resistance was observed, with the first signs of mould being microscopically identified only after 28 days of exposure in a wet environment.

On the other hand, the microscopic analysis of the samples showed, in general, quantitative growth in mould from one evaluation to the next, with some of the samples being classified in the next class of fungal growth rating, i.e., class 2. Due to the shape of the material, most of which is loose, it is very difficult to assess the mould surface area proportionally, but for all these samples classified in fungal growth rating class 2, a material performance category of minimum 2 can be assumed, i.e., the material can be assessed as containing nutrients that allow mould growth.

Of the 10 materials analysed, none was identified as having a behaviour compatible with maintaining fungal growth rating class 0 (no sign of growth on microscopic examination) and performance category in terms of nutrient content conducive to the growth of microorganisms 0 (not a nutrient medium for microorganisms—inert or fungistatic). Therefore, it is considered that for all the cases analysed an antifungal treatment is required or, if this treatment existed in the production process, it is insufficient. Also, based on the results, it is considered necessary that, when these products are put into operation, the technology should provide for the use of specific protections to reduce the contact of the material with the humidity in the environment.

#### 3.3.2. Resistance of Heat-Insulating Materials under Conditions Contaminated with Micro-Organisms

**Figure 7:**
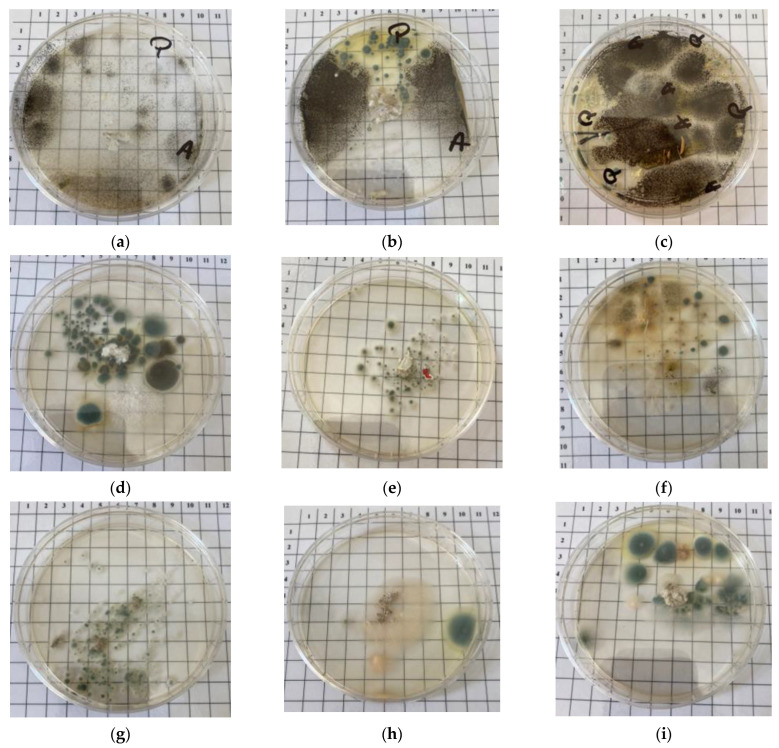
Development of inoculated and native microflora on culture media (*A*—*Aspergillus niger*; *P*—*Penicillium notatum*). (**a**) P3 (cellulose acetate, waste cigarette filter manufacture, waste cigarette paper and waste aluminised paper)—inoculated, (**b**) P8 (cellulose from waste paper, producer 2)—inoculated, (**c**) P10 (composite based on sheep wool, recycled PET fibres and cellulosic fibres for the textile industry)—inoculated, (**d**) P2 (cellulose acetate, cigarette filter manufacturing waste and cigarette paper waste)—native, (**e**) P3 (cellulose acetate, waste cigarette filter manufacture, waste cigarette paper and waste aluminised paper)—native, (**f**) P5 (wood fibres)—native, (**g**) P6 (cellulose from cardboard waste)—native, (**h**) P7 (cellulose from waste cardboard, poor processing, inhomogeneous product)—native, (**i**) P8 (cellulose from waste paper, producer 2)—native.

Both cultivation techniques showed an interesting behaviour of the tested insulation. The test for native microflora revealed the growth of fungi on all the insulations, regardless of the type (Table 8 and shown in Figure 7). Sample P1 showed class 2 growth of native fungi, with the development of small colonies around the insulation. The same class was established in inoculated conditions, both fungal species showing growth but with colonies under 1 cm in diameter. Sample P2 permitted the development of numerous colonies around the insulation, with the native microflora established as class 4, but in controlled inoculation, this insulation maintained the colonies of both species under 1 cm in diameter. The third type of insulation showed multiple small colonies (class 3), which developed as native microflora. In controlled inoculation, this insulation did not permit the growth of *Penicillium notatum*, unlike *Aspergillus niger* which proliferated on media. Insulation P4 showed abundant mycelium growth (native microflora), but blocked the development of *A. niger*. The same insulation restricted the development of *P. notatum* as a long and thin colony. The P5 insulation did not permit the development of either inoculated species but was sensitive to the native microflora test—where multiple confluent colonies were observed. The same results were obtained for insulations P6 and P7 in the native test, both showing high dimension colonies. The inoculation test for both insulations showed medium resistance to fungal contaminants, with small colonies developed—under 1 cm in diameter. Insulation P8 was considered as class 4 resistant to native microflora test, compared to insulations P9 and P10, which were considered as class 5. All three insulations showed well-developed colonies emerged from the insulation up to the complete coverage of fungal mycelium. In terms of controlled inoculation, all three insulations showed a different reaction. Insulation P8 was affected by both inoculated species, each having an equal share on culture media. Both P9 and P10 showed a resistance to *P. notatum*, but no resistance to *A. niger*.

### 3.4. Benefits and Challenges of Loose-Fill Insulation in Construction

Loose-fill insulation has become popular due to its installation facility, cost-effectiveness, and suitability for insulating rather complex spaces. For example, insulating the voids between roof rafter structures is greatly accelerated when blowing loose-fill insulation, compared to the more labor-intensive installation of traditional insulation [57] that presents as rolls or boards. A remarkable use of loose-fill insulation is for house attics, a process proven to be quick and that minimises material waste [71]. The installation process of this thermal insulation method is simple, which helps reduce labour costs and makes it cost-effective overall. These features are appealing to construction companies and other relevant stakeholders. As a result of the specific behaviour and efficiency of eco-innovative thermal insulation materials, in the specialised literature, some reviews indicate the possibility of use, for example, for bedroom wall insulation (which could provide benefits of even 5–30% in terms of energy consumption) as indicated by [72,73]. Other studies [74,75] suggest that this insulation material would be suitable for insulating buildings with air conditioning and humidity control systems.

Additionally, incorporating loose-fill insulation aligns with sustainable and environmentally conscious construction practices. However, sourcing and producing these insulating materials locally is crucial to meet the eco-friendly goals. This approach significantly reduces both environmental and transportation costs associated with the product. By doing so, we can minimise transportation’s carbon footprint while also strengthening local economies. It is important to note that produced loose-fill insulating materials may have unique properties due to variations in local resources [76,77] and manufacturing techniques [78,79]. One target of this research is to present some differences, where similar products or identical raw materials used in conjunction with different technologies yield notably different results. Consequently, conducting thorough testing of these products is vital to ensure their quality, performance, and safety. Thorough testing can identify the product’s characteristics, such as thermal conductivity and density, allowing these materials to be used where they perform best.

## 4. Conclusions

The aim of this work was to analyse the performance and behaviour of 10 types of thermal insulation materials available on the building materials market in the “niche” area, given their origin from agro-industrial waste raw materials. The experimental requirements followed three directions:

1. Impact on the energy consumption of buildings and indoor air quality through their thermal insulation performance, quantified by the thermal conductivity coefficient determined on dry material, and on conditioned material at a temperature of 23 °C ± (0.25 ÷ 0.3) °C and a relative air humidity (RH) of (50 ± 1)%.
2. Impact on indoor air quality through its ability to regulate humidity through the phenomenon of moisture sorption-desorption.
3. Impact on indoor air quality in terms of contamination by micro-organisms, through an analysis of the risk of mould growth and resistance to mould attack by *Aspergillus niger* and *Penicillium notatum*.

Based on the experimental results obtained, the following can be appreciated:

- In terms of thermal performance, the 10 types of materials analysed correspond to the criteria imposed by the intended field of use, with thermal conductivity coefficients comparable to those of common insulating materials;
- The coefficient of thermal conductivity is influenced by a variety of factors. These include, evidently, the type of raw material used in the production of the material, the process by which it is manufactured, its density, its moisture content, and the temperature at which the test is conducted. Typically, the presence of moisture has a negative impact on thermal performance. An increase in density, usually achieved by compressing the material, tends to reduce thermal conductivity for materials with uniform distributed air-gaps. Our experimental results show that product P3 (composite containing cellulose acetate, waste cigarette filter manufacture, waste cigarette paper and waste aluminised paper), as well as P8 (produced from waste paper by producer 2), perform best at their natural densities. For these materials, the most effective and practical thermal conductivity coefficient is achieved without altering their natural density;
- In terms of sorption-desorption capacity, a relative consistency of sorption was observed, with a slightly increasing trend, up to and including RH 75%, followed by an amplification of the phenomenon in the area of RH >75%. The most obvious intensification of sorption was recorded in the case of the thermal insulation material made from wood fibres (P5) and the one made from cellulose from waste cardboard, poor processing, inhomogeneous product (P7). The desorption curves show a lower capacity for loss than for accumulation of atmospheric moisture, i.e., following desorption, a residual amount of the adsorbed water remains in the material. Therefore, the materials analysed showed a certain capacity to contribute to the regulation of indoor air humidity, but the residual water indicated by the desorption curves not only does not contribute to the regulation of indoor air humidity, but may even lead to the creation of an environment favourable to mould growth;
- In terms of resistance to mould growth in a high humidity environment, the experimental results showed that all the materials analysed are at risk of mould growth after a longer or shorter period of exposure to a humid atmosphere, depending on the nature of the material;
- Each type of tested insulation carries a specific microflora, which can grow in optimum conditions from small-isolated colonies around the material up to complete cover of it. The artificial inoculation of insulation with two fungal species revealed a gradual resistance to colonisation from zero growth up to complete cover of one or both species;
- For none of the analysed materials, the preservation of the fungal growth rating class 0 and the performance category in terms of nutrient content conducive to the growth of microorganisms 0 was not identified; therefore a more effective antifungal treatment is required.

The scientific contribution of this study can be summarised as follows:

- It analyses a significant number of thermal insulation materials made by recycling agro-industrial waste, materials that are present in a construction materials market that is still underdeveloped;
- The experimental analysis is interdisciplinary, considering several aspects: thermal insulation performance both under dry conditions and under conditions of conditioning at a temperature of 23 °C ± (0.25 ÷ 0.3) °C and RH (50 ± 1)%; thermal insulation performance both under natural density conditions and for several degrees of compaction; the capacity for moisture absorption/desorption depending on the relative humidity of the air and resistance to mould action;
- It draws attention to weak points, particularly the need for antifungal treatments;
- Although there are some weak points, opportunities for improvement are identified and the possibility of obtaining materials that simultaneously respond to two pressing needs is highlighted: environmental protection through waste recycling and reducing energy consumption through the thermal insulation of spaces dedicated to human activities.

## Figures and Tables

**Table 1:** Coding and characterisation of thermal insulation materials.

Sample Code	Density in Natural State (kg/m^3^)	Marketing Method	Installing	Raw Material for Production
P1	21.2	bulk	inside the building, loose between rigid panels	cellulose acetate, cigarette filter manufacturing waste
P2	31.7			cellulose acetate, cigarette filter manufacturing waste and cigarette paper waste
P3	38.9			cellulose acetate, waste cigarette filter manufacture, waste cigarette paper and waste aluminised paper
P4	35.1			cellulose from waste paper, producer 1
P5	23.4			wood fibres
P6	55.8			cellulose from cardboard waste
P7	125.5			cellulose from waste cardboard, poor processing, inhomogeneous product
P8	48.0			cellulose from waste paper, producer 2
P9	98.2			rice husk waste
P10	20.7	non-woven mattress	inside the building, on wooden frames	composite based on sheep wool, recycled PET fibres and cellulosic fibres for the textile industry

**Table 2:** Evaluation of fungal growth and product performance.

Class	Evaluation of Fungal Growth	Category	Product Performance Estimation
Class 0	No sign of growth on microscopic examination	0	The material is not a nutrient medium for microorganisms (it is inert or fungistatic).
Class 1	Growth invisible to the naked eye but clearly visible under a microscope	1	The material contains few nutrients or is so little contaminated that it allows very little growth.
Class 2	Increase visible to the naked eye, covering up to 25% of the test area	2–3	The material is not resistant to attack by micro-organisms; it contains nutrients that allow them to grow.
Class 3	Increase visible to the naked eye, covering up to 50% of the test area.		
Class 4	Increase visible to the naked eye, covering more than 50% of the test area.		
Class 5	Strong growth covering the entire test surface.		

**Table 3:** Dependence of thermal conductivity coefficient on dry material density.

Dry Material	Sample Temperature 10 °C		Sample Temperature 25 °C		Sample Temperature 40 °C	
	λ = f(Density)	R^2^	λ = f(Density)	R^2^	λ = f(Density)	R^2^
P1	y = 0.0047x^2^ − 0.4963x + 46.103	0.95	y = 0.0051x^2^ − 0.5519x + 49.817	0.92	y = 0.0058x^2^ − 0.642x + 54.771	0.93
P2	y = 0.0038x^2^ − 0.4432x + 48.245	0.96	y = 0.0045x^2^ − 0.5431x + 53.096	0.98	y = 0.0045x^2^ − 0.5567x + 56.44	0.98
P3	y = 0.5337x + 49.509	0.99	y = 0.5382x + 52.702	1.00	y = 0.5063x + 58.577	0.99
P4	y = 0.0016x^2^ − 0.1591x + 43.732	0.99	y = 0.0016x^2^ − 0.1727x + 46.485	0.95	y = 0.0021x^2^ − 0.2479x + 50.885	0.99
P5	y = 0.0037x^2^ − 0.3886x + 47.437	0.97	y = 0.0049x^2^ − 0.516x + 52.508	0.98	y = 0.0052x^2^ − 0.5732x + 56.907	0.98
P6	y = 0.0027x^2^ − 0.4649x + 66.824	1.00	y = 0.0032x^2^ − 0.5704x + 74.574	1.00	y = 0.0037x^2^ − 0.6797x + 83.456	1.00
P7	y = 0.0007x^2^ − 0.1633x + 64.8	1.00	y = 0.0007x^2^ − 0.1699x + 69.025	1.00	y = 0.001x^2^ − 0.2772x + 82.96	1.00
P8	y = 0.0016x^2^ − 0.1468x + 45.449	1.00	y = 0.0017x^2^ − 0.1745x + 48.743	1.00	y = 0.0018x^2^ − 0.1945x + 51.474	1.00
P9	y = 0.0074x^2^ − 1.6584x + 136.88	0.99	y = 0.0135x^2^ − 3.0344x + 216.08	0.98	y = 0.0099x^2^ − 2.1941x + 171.64	0.98
P10	y = 0.0018x^2^ − 0.3168x + 44.139	0.95	y = 0.0021x^2^ − 0.3684x + 48.13	0.96	y = 0.0025x^2^ − 0.4435x + 53.464	0.96

**Table 4:** Dependence of the thermal conductivity coefficient on the density of the conditioned material at 23 °C ± (0.25 ÷ 0.3) °C and RH (50 ± 1)%.

Material Conditioned at 23 °C ± (0.25 ÷ 0.3) °C and RH (50 ± 1)%	Sample Temperature 10 °C		Sample Temperature 25 °C		Sample Temperature 40 °C	
	λ = f(Density)	R^2^	λ = f(Density)	R^2^	λ = f(Density)	R^2^
P1	y = 0.0045x^2^ − 0.4872x + 46.85	0.97	y = 0.0047x^2^ − 0.5034x + 50.052	0.97	y = 0.0051x^2^ − 0.5706x + 55.084	0.98
P2	y = 0.003x^2^ − 0.3775x + 47.308	0.92	y = 0.005x^2^ − 0.5988x + 55.32	0.99	y = 0.0041x^2^ − 0.5128x + 56.485	0.98
P3	y = 0.489x + 50.809	0.99	y = 0.5024x + 54.4	0.99	y = 0.5481x + 56.682	0.99
P4	y = 0.0031x^2^ − 0.3843x + 49.652	0.95	y = 0.0021x^2^ − 0.2408x + 49.443	0.93	y = 0.0018x^2^ − 0.2059x + 50.745	0.99
P5	y = 0.0039x^2^ − 0.38x + 46.878	0.99	y = 0.0039x^2^ − 0.4263x + 52.667	0.95	y = 0.0036x^2^ − 0.4167x + 55.488	0.96
P6	y = 0.0042x^2^ − 0.7433x + 78.387	1.00	y = 0.0032x^2^ − 0.5817x + 76.898	1.00	y = 0.0036x^2^ − 0.7015x + 87.968	1.00
P7	y = 0.0008x^2^ − 0.1617x + 64.769	1.00	y = 0.0005x^2^ − 0.0989x + 65.97	1.00	y = 0.0008x^2^ − 0.1919x + 77.077	1.00
P8	y = 0.0022x^2^ − 0.2147x + 46.37	1.00	y = 0.0025x^2^ − 0.2451x + 51.515	1.00	y = 0.0037x^2^ − 0.4469x + 62.038	1.00
P9	y = 0.006x^2^ − 1.4232x + 131.05	0.91	y = 0.0082x^2^ − 1.9444x + 165.66	0.99	y = 0.0094x^2^ − 2.2358x + 184.11	0.99
P10	y = 0.002x^2^ − 0.3411x + 45.721	0.98	y = 0.0021x^2^ − 0.3837x + 50.267	0.98	y = 0.0022x^2^ − 0.4147x + 55.211	0.96

**Table 5:** Calculated density (kg/m^3^) for which the thermal conductivity coefficient (W/mK) is minimum, for dry material, i.e., conditioned at 23 °C ± (0.25 ÷ 0.3) °C and RH (50 ± 1)%.

Material	Dry						Conditioned at 23 °C ± (0.25 ÷ 0.3) °C and RH (50 ± 1)%					
	10 °C		25 °C		40 °C		10 °C		25 °C		40 °C	
	kg/m^3^	W/mK	kg/m^3^	W/mK	kg/m^3^	W/mK	kg/m^3^	W/mK	kg/m^3^	W/mK	kg/m^3^	W/mK
P1	52.8	0.0330	54.1	0.0345	55.3	0.0370	54.1	0.0337	53.6	0.0366	55.9	0.0391
P2	58.3	0.0353	60.3	0.0367	61.9	0.0392	62.9	0.0354	59.9	0.0374	62.5	0.0405
P3 *	-	-	-	-	-	-	-	-	-	-	-	-
P4	49.7	0.0398	54.0	0.0418	59.0	0.0436	62.0	0.0374	57.3	0.0425	57.2	0.0449
P5	52.5	0.0372	52.7	0.0389	55.1	0.0411	48.7	0.0376	54.7	0.0410	57.9	0.0434
P6	86.1	0.0468	89.1	0.0492	91.9	0.0522	88.5	0.0455	90.9	0.0505	97.4	0.0538
P7 *	-	-	-	-	-	-	-	-	-	-	-	-
P8	45.9	0.0421	51.3	0.0622	54.0	0.0462	48.8	0.0411	49.0	0.0455	60.4	0.0485
P9	112.1	0.0440	112.4	0.0456	110.8	0.0501	118.6	0.0467	118.6	0.0504	118.9	0.0512
P10	88.0	0.0302	87.7	0.0320	88.7	0.0338	85.3	0.0312	91.4	0.0327	94.3	0.0357

* The calculation of corresponding values for materials P3 and P7 was not performed due to the monotonically increasing nature of the modelling functions. This means that the lowest thermal conductivity coefficient value is found at the material’s natural density, specifically, the density under its own weight.

**Table 6:** Typical thermal conductivity values for insulation materials.

Material	Density (kg/m^3^)	Thermal Conductivity (W/mK)	Material	Density (kg/m^3^)	Thermal Conductivity (W/mK)
EPS foam	30	0.0375	P3	38.9	0.070
Rock wool	60	0.040	P4	35.1	0.040
Polyurethane rigid foam	30	0.032	P5	24.3	0.040
Cork slab	150	0.049	P6	55.8	0.049
Cellulose fibre	50	0.040	P7	125.5	0.055
Wood wool	180	0.070	P8	48.0	0.042
P1	21.2	0.038	P9	106.0	0.045
P2	31.7	0.038	P10	20.7	0.039

Note: For the materials analysed (P1–P10), the thermal conductivity coefficient, λ_10,ct_, determined for the dry material, at natural density (without settling), determined at a sample average temperature of 10 °C, was given.

**Table 7:** Fungal growth rating class and performance category of the material in terms of nutrient content conducive to the growth of microorganisms.

Sample Code	P1	P2	P3	P4	P5	P6	P7	P8	P9	P10
	Fungal Growth Rating Class/Performance Category									
5 days exposure	0/0	0/0	1/1	1/1	0/0	0/0	0/0	0/0	0/0	0/0
10 days exposure	0/0	1/1	1/1	1+/1	0/0	1/1	0/0	1/1	1/1	0/0
15 days exposure	0/0	1+/1	1+/1	1+/1	1/1	1+/1	1/1	1+/1	1+/1	1/1
20 days exposure	0/0	1+/1	1+/1	1+/1	1+/1	1+/1	1+/1	1+/1	1+/1	1+/1
25 days exposure	0/0	1+/1	1+/1	1+/1	1+/1	1+/1	1+/1	1+/1	1+/1	2/2
30 days exposure	1/1	2/2	2/2	2/2	1+/1	1+/1	2/2	2/2	2/2	2+/2

**Table 8:** Fungal growth rating class and performance category of the material under growing medium conditions.

Sample		P1	P2	P3	P4	P5	P6	P7	P8	P9	P10
Native microflora		2	4	3	3/4	4	4	4	4	5	5
Inoculation test	*Penicillium notatum*	2	2	-	2	-	2	2	4/5	2	2
	*Aspergillus niger*	2	2	4	-	-	2	2	4/5	5	5

## Data Availability

The data presented in this study are available on request from the corresponding authors.
